# Association between local inflammation and breast tissue age-related lobular involution among premenopausal and postmenopausal breast cancer patients

**DOI:** 10.1371/journal.pone.0183579

**Published:** 2017-08-28

**Authors:** Mirette Hanna, Isabelle Dumas, Michèle Orain, Simon Jacob, Bernard Têtu, François Sanschagrin, Alexandre Bureau, Brigitte Poirier, Caroline Diorio

**Affiliations:** 1 Oncology Research Unit, CHU de Québec Research Center, Université Laval, Québec, Québec, Canada; 2 Department of Social and Preventive Medicine, Cancer Research Center, Université Laval, Québec, Québec, Canada; 3 Department of Molecular Biology, Medical Chemistry and Pathology, Cancer Research Center, Université Laval, Québec, Québec, Canada; 4 Service of Molecular Biology, Medical Chemistry and Pathology, Hôpital Saint-Sacrement, CHU de Québec, Université Laval, Québec, Québec, Canada; 5 Centre des Maladies du Sein Deschênes-Fabia, Hôpital du Saint-Sacrement, Québec, Québec, Canada; 6 Centre de Recherche de l’Institut Universitaire en Santé Mentale de Québec, Université Laval, Québec, Québec, Canada; 7 Department of Surgery, Cancer Research Center, Université Laval, Québec, Québec, Canada; University of Arkansas for Medical Sciences, UNITED STATES

## Abstract

Increased levels of pro-inflammatory markers and decreased levels of anti-inflammatory markers in the breast tissue can result in local inflammation. We aimed to investigate whether local inflammation in the breast tissue is associated with age-related lobular involution, a process inversely related to breast cancer risk. Levels of eleven pro- and anti-inflammatory markers were assessed by immunohistochemistry in normal breast tissue obtained from 164 pre- and postmenopausal breast cancer patients. Involution status of the breast (degree of lobular involution and the predominant lobule type) was microscopically assessed in normal breast tissue on hematoxylin-eosin stained mastectomy slides. Multivariate generalized linear models were used to assess the associations. In age-adjusted analyses, higher levels of pro-inflammatory markers IL-6, TNF-α, CRP, COX-2, leptin, SAA1 and IL-8; and anti-inflammatory marker IL-10, were inversely associated with the prevalence of complete lobular involution (all *P*≤0.04). Higher levels of the pro-inflammatory marker COX-2 were also associated with lower prevalence of predominant type 1/no type 3 lobules in the breast, an indicator of complete involution, in age-adjusted analysis (*P* = 0.017). Higher tissue levels of inflammatory markers, mainly the pro-inflammatory ones, are associated with less involuted breasts and may consequently be associated with an increased risk of developing breast cancer.

## Introduction

Many cellular processes controlling normal breast maturation-regression have also been found to be involved in cancer development. The immune system has been linked to normal mammary gland development as well as breast carcinogenesis [1–3]. Continuous stimulation of the immune system by persistent tissue injury can create a state of chronic local inflammation. Chronic local inflammation provides signals that can alter the proliferation, differentiation and apoptosis of breast cells [4–7]. Mammary cells respond to these signals by maintaining cellular proliferation, accumulating reactive oxygen species and enhancing the production of more inflammatory markers. Chronic local inflammation can thus be expected to deregulate the physiological breast maturation-regression steps.

Different breast maturation-regression stages were firstly described by Russo and Russo [8]. During the stage of lobular maturation, epithelium forming undifferentiated type 1 lobules (<12 acini) evolves into type 2 lobules (12–80 acini) which will develop into more complex type 3 lobules (>80 acini) under hormonal stimulation. As women age, breast lobules undergo age-related lobular involution (ARLI). ARLI is the physiologic breast atrophy characterised by regression of the breast to an earlier stage of development; gradual replacement of epithelium and stroma by collagen, and then eventually by adipose tissue, and regression of type 2 and 3 lobules to type 1 lobules [9].

The ARLI was found to be associated with lower breast cancer (BC) risk. Women having involuted breasts have lower BC risk compared to those with partial or no involution [10–12]. The ARLI begins around the age of 40 and accelerates after menopause; involution may remain incomplete at age 60 in almost 80% of women [13]. As of yet, genetic and environmental factors that may explain inter-individual variations in the age of initiation of ARLI, the rate at which this process advances, have not been thoroughly examined in formal epidemiological studies. Recently, higher circulating levels of IGF-I levels were inversely associated to the TDLU counts, a marker of the degree of lobular involution (*P*-trend = 0.009) among 73 postmenopausal women diagnosed with benign breast diseases in multivariate analysis [14]. Moreover, higher circulating levels of unconjugated estradiol, 2-hydroxyestrone, and 4-hydroxyestrone were associated with less involuted breasts among 94 premenopausal women undergoing diagnostic breast biopsy in adjusted analysis for several confounding factors not including the phase of the menstrual cycle (*P*-trend = 0.03, *P*-trend = 0.04 and *P*-trend = 0.04, respectively) [15]. Of note, among the 94 women, 31 women (33%) were in the follicular phase of the menstrual cycle, 25 women (26.6%) in the periovulatory phase and 38 women (40.4%) in the luteal phase. Similarly, higher circulating levels of estradiol and 16α-hydroxyestrone were inversely associated with the ARLI (*P*-trend = 0.04 and *P*-trend = 0.02, respectively) among 92 postmenopausal women undergoing diagnostic breast biopsy [15].

Interestingly, a study found that lobulitis, defined as the presence of immune cells, namely helper T cells (CD4), cytotoxic T cells (CD8), B cells (CD20) and leukocytes (CD45), found in lobules, varied by the degree of lobular involution assessed in 109 lobules in the normal breast tissue of healthy women [16]. To our knowledge, the association between the expression of inflammatory markers and the ARLI has never been explored before. Therefore, we conducted a cross-sectional study of 164 pre- and postmenopausal BC patients to investigate the association of pro- and anti-inflammatory markers in normal breast tissue with the ARLI expressed in the degree of lobular involution and the predominant lobule type. We considered eight pro-inflammatory markers [interleukin 6 (IL-6), tumor necrosis factor-α (TNF-α), C-reactive protein (CRP), cyclooxygenase 2 (COX-2), leptin, serum amyloid A1 (SAA1), interleukin 8 (IL-8) and signal transducers and activators of transcription 3 (STAT3)] and three anti-inflammatory markers [transforming growth factor-β (TGF-β), interleukin 10 (IL-10) and lactoferrin]. We hypothesized that higher prevalence of complete involution or predominant type 1/no type 3 lobules would be observed among women with lower levels of pro-inflammatory markers and higher levels of anti-inflammatory markers in breast tissue.

## Materials and methods

### Study population and data collection

Selection of the study population has been described elsewhere [17]. Briefly, women diagnosed with unilateral BC at the Centre des Maladies du Sein Deschênes-Fabia from January 2011 through April 2012, having had a total or partial mastectomy, aged <70 years at the time of recruitment, and having received a mammogram within the six months preceding the diagnosis were eligible for this study. Women were excluded if they were currently pregnant, had a history of breast surgeries, had received neoadjuvant therapies, or had a previous diagnosis of cancer other than non-melanoma skin cancer. A total of 164 women were considered and thereafter provided signed informed consent. Study protocol was approved by the Research Ethics Board of the Centre Hospitalier Universitaire de Québec, Québec (QC), Canada.

Laterality and location of BC were identified by review of pathology reports. Information on known and suspected BC risk factors were collected during personal and telephone interviews; anthropometric measures (weight, height and waist and hip circumferences), reproductive data (age at menarche, age of first pregnancy and number of full-term pregnancies), history of breastfeeding, use of hormonal contraceptives or hormonal replacement therapy, breast biopsies, smoking, regular use of multivitamins or anti-inflammatory drugs, alcohol intake and family history of BC. Postmenopausal women were defined as those who had natural or surgically induced amenorrhea for 12 months or more (hysterectomy with bilateral oophorectomy), or those aged 53 or 55 years or more for smokers and non-smokers, respectively, and had hysterectomy without bilateral oophorectomy. Other women were considered premenopausal. Menopausal status could not be determined in eleven women aged <53 years. They were added to premenopausal women (n = 71) in further analyses as their ARLI were similar to that of premenopausal women.

### Assessment of inflammatory markers

#### Tissue microarray construction

For all participants, two pathologists (BT and SJ), blinded to women’s information, identified zones of morphologically normal terminal ductal lobular units (TDLUs) on the hematoxylin-eosin (H&E) stained slides located at >1.0 cm from the tumor. Next, six cores (1.0 mm in diameter each) were extracted from the corresponding zones on the formalin-fixed paraffin embedded (FFPE) mastectomy blocks and randomly arrayed on recipient paraffin tissue microarray (TMA) blocks. Every participant was represented twice on a TMA block. Furthermore, each of the three BC cell lines (MCF-7, MDA-231 and SKBR-3) was placed in duplicate on every TMA block to serve as internal positive controls during the immunohistochemistry staining. In addition, a TMA block containing normal (tonsils, breast, thymus, colon and liver), inflammatory (colon) and malignant (breast, colon, liver, lung, ovary, prostate and kidney) human tissues, was constructed to serve as an external control. All TMAs were constructed using the tissue punch arrayer (Beecher Instruments^®^ Tissue Microarray Technology, Estigen, Sun Prairie, WI, USA).

### Immunohistochemistry staining

TMAs serial cut sections (4 μm in thickness) were performed. The first and last sections from each block were H&E stained for histological examination. Other sections were stained according to standardized immunohistochemistry protocol for eleven pro- and anti-inflammatory markers. The choice of inflammatory markers was based on 1) their expression in normal breast tissue in detectable amount, 2) their documented effect on mammary cells behaviour [4–7], and 3) the commercial availability of validated antibodies that perform robustly in FFPE tissue. Heat Induced Epitope antigen Retrieval (HIER) was performed for COX-2, SAA1, STAT3, IL-8, TGF-β, IL-10 and lactoferrin stained slides, using prewarmed citrate buffer (pH 6.0) for 12 min. Slides were incubated with primary antibodies at the optimum concentration and duration as established by a prior titration assay; anti-IL-6 (mouse mAb, sc-130326; Santa Cruz Biotechnology, 1:150 for 1 hour), anti-TNF-α (mouse mAb, (52B83); Santa Cruz Biotechnology, 1:50 for 2 hours), anti-CRP (rabbit mAb, 1568–1; Epitomics, 1:100 for 1 hour), anti-COX-2 (mouse mAb, 358200; Invitrogen, 1:100 for 1 hour), anti-Leptin (rabbit pAb, (A-20) sc842; Santa Cruz Biotechnology, 1:200 for 1 hour), anti-SAA1 (mouse mAb, AT375a; ABGENT, 1:50 for 1 hour), anti-pSTAT-3 (rabbit mAb, 2236–1; Epitomics, 1:250 overnight incubation), anti-IL-8 (mouse mAb, 60141-1-Ig; Proteintech Group, 1:100 for 2 hours), anti-TGF-β (mouse mAb, MCA797; AbDserotec, 1:500 for 1 hour), anti-IL-10 (rat mAb, MCA2250; AbDserotec, 1:200 overnight incubation) and anti-lactoferrin (rabbit mAb, 3271–1; Epitomics, 1:100 for 1 hour). Corresponding secondary antibody were used, either the anti—mouse antibody (DAKO, EnVision+ System-HRP (DAB), Bucks, UK) or the rat, rabbit and guinea pig antibody (IDetect Super Stain HRP Polymer Kit, Ontario, Canada). Stained slides were scanned with NanoZoomer 2.0-HT (Hamamatsu, Bridgewater, NJ, USA) to generate high-resolution images at 20x magnification. Positive and negative control slides from the control TMA block were processed similarly in each staining run. More details regarding the immunostaining procedures were provided elsewhere [18].

### Interpretation of immunostaining

**Fig 1:**
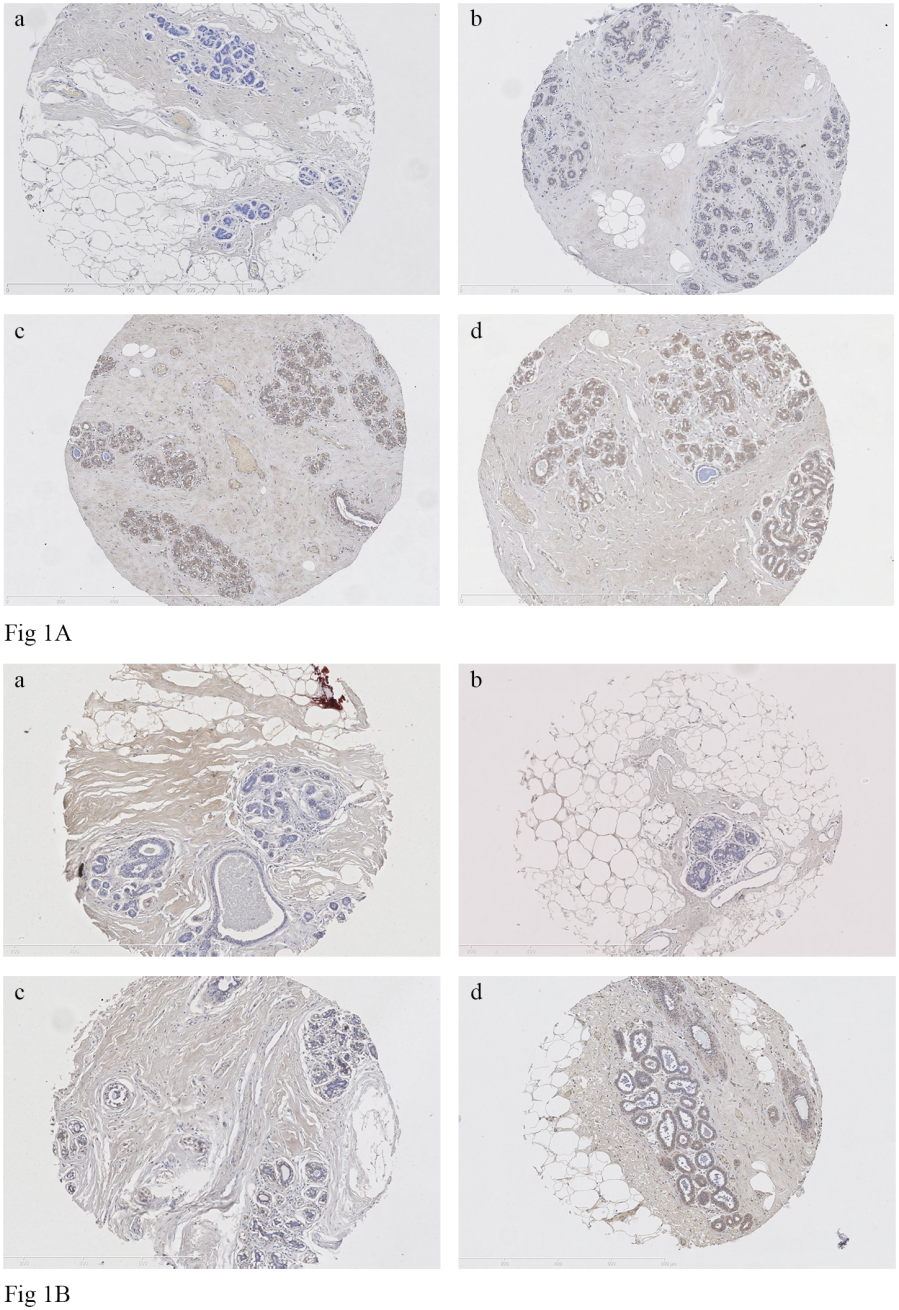
**Fig 1A**. Representative immunohistochemistry staining for the intensity of the protein levels of TNF-α (mouse mAb, (52B83); Santa Cruz Biotechnology) in TMA of normal breast tissue (magnification 10x): a) negative staining, b) weak staining, c) moderate staining and d) strong staining. **Fig 1B**. Representative immunohistochemistry staining for the extent of the protein levels of TNF-α (mouse mAb, (52B83); Santa Cruz Biotechnology) in TMA of normal breast tissue (magnification 10x): a) 0, 0%; b) 1, 1–9%; c) 2, 10–50% and d) 3, >50% of cells stained positive for the TNF-α.

For all markers, the intensity and extent of immunostaining of the epithelial component of the cores present on TMA slides were visually evaluated by one blinded reader (MH) For COX-2, immunostaining was assessed separately in both the epithelium and stroma. Staining intensity was scored 0–3 corresponding to negative, weak, medium or strong staining, respectively. The extent was expressed as the proportion of positively stained cells, scoring 0, 1, 2, or 3 for 0%, 1–9%, 10–50%, and >50% of cells stained positive, respectively. The immunostaining was individually evaluated in each of the 1–6 cores judged interpretable on the TMA stained slides for one woman. Then, the median of the intensity and the extent of all cores were estimated (representative of different categories of intensity and extent of immunostaining are provided in Fig 1A and 1B, respectively). Next, the quick score was obtained by multiplying the median of the intensity (0–3) by that of the extent (0–3) and then dichotomized into low vs. high expression using the median of each marker as a cut-off. We also estimated the pro- and anti-inflammatory score by summing the original scale quick score for the eight pro-inflammatory or the three anti-inflammatory markers, respectively. Five randomly selected TMA slides were re-evaluated by the same reader and a second reader to assess the reproducibility of the analysis (kappa = 0.75 (95% CI = 0.64–0.86) and 0.74 (95% CI = 0.63–0.84) for intra- and inter-observer agreement, respectively).

### Age-related lobular involution

The study pathologists (BT and SJ) selected one H&E stained slide containing normal breast tissue located at least 1.0 cm from the tumor for each woman. Involution status verified on a single tissue specimen represents the involution status of both breasts [19].

**Fig 2:**
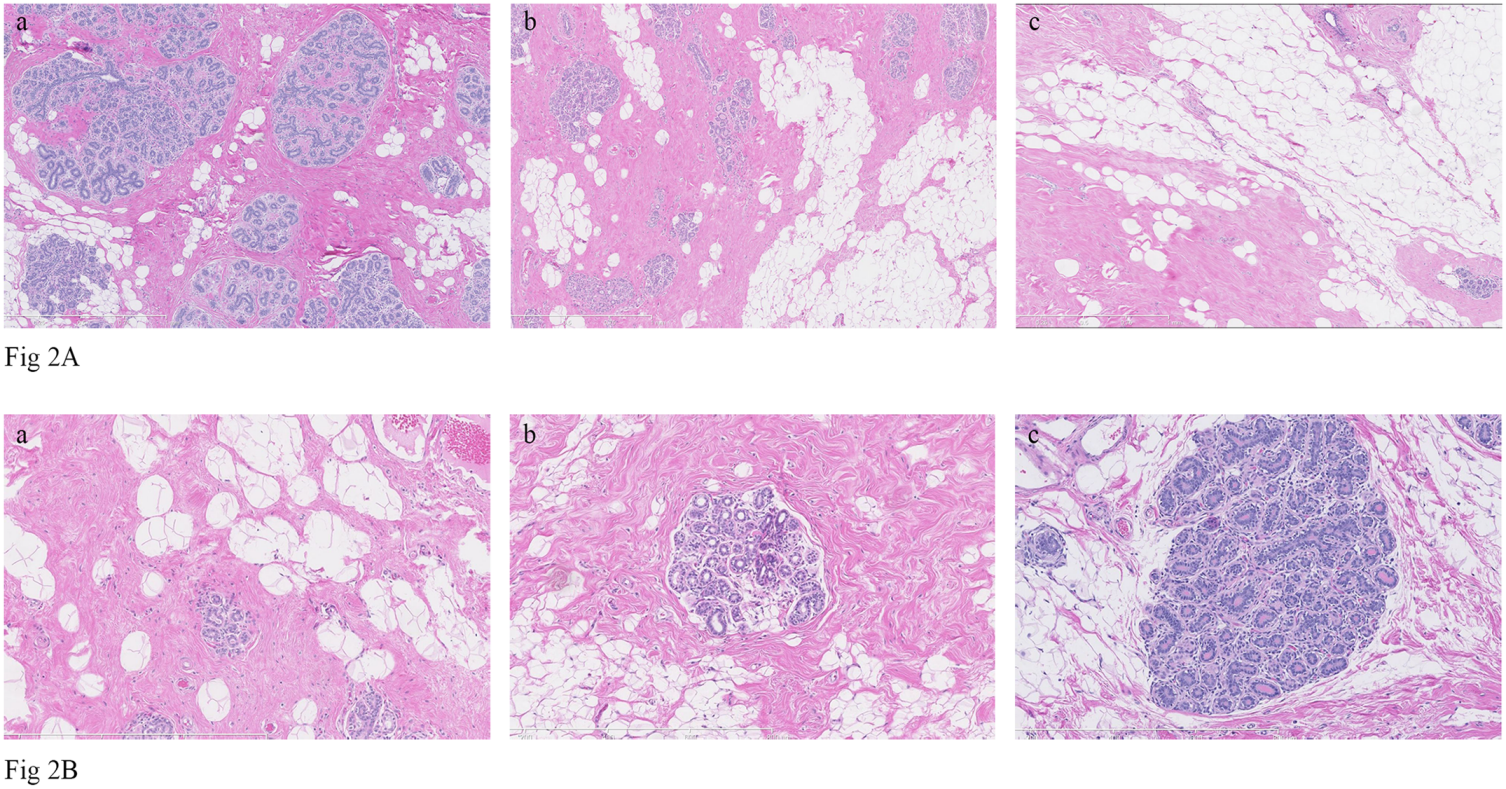
**Fig 2A**. Representative hematoxylin-eosin staining of different categories of degree of lobular involution in normal breast tissue (magnification 5x): a) no involution (0%TDLUs involuted), b) partial involution (1–74% TDLUs involuted) and c) complete involution (≥75% TDLUs involuted). **Fig 2B**. Representative hematoxylin-eosin staining of different lobule types (magnification 10x): a) type 1 lobule (<12 acini), b) type 2 lobule (12–80 acini) and type 3 lobule (>80 acini).

We considered two measures of the ARLI previously shown to be associated with BC risk: the degree of lobular involution, a qualitative measure, and the predominant lobule type, a quantitative measure [10, 11, 20]. The degree of lobular involution was classified according to the percentage of involuted TDLUs having few to several small acini and flattened inconspicuous acinar epithelium with fibrosis of specialized intralobular stroma [10]. The degree of lobular involution was classed under one of three categories: no, partial and complete for 0, 1–74 and >75% involuted TDLUs, respectively [10]. In addition, the type, and the proportion of each type of lobule among all lobules present on the selected slide (type 1, 2 and 3 lobules for <12, 12–80 and >80 acini, respectively) [8] as well as the predominant type were identified. The predominant type had to constitute ≥60% of the total number of lobules [21]. Lobules containing atypical lobular or ductal hyperplasia, sclerosing adenosis or large cyst were excluded. Consequently, slides were grouped into two categories of each measure of ARLI based on BC risk [11, 20]: no or partially involuted for the degree of lobular involution vs. completely involuted and predominant type 1/no type 3 lobules vs. other lobule types for the predominant lobules type (representative H&E staining of different categories of degree of lobular involution and different lobule types are presented in Fig 2A and 2B, respectively). The degree of lobular involution and the predominant lobule type were blindly assessed by one reader (MH) for all women. For validation of the two measures, a total of 40 randomly selected cases were re-evaluated by two different readers (BT and SJ). The two measures were highly reproducible as previously reported (kappa = 0.71 and 0.88 for the degree of lobular involution and the predominant lobule type, respectively) [17].

### Statistical analyses

Our objective was to evaluate whether the breast involution status, either the degree of lobular involution or the predominant lobule type, varied by levels of inflammatory proteins in normal breast tissue. So, we considered inflammatory marker expression using a two-category quick score, a pro-inflammatory or an anti-inflammatory score as the explanatory variables. The low expression category of the two-category quick score was considered as the referent category. The pro- or anti-inflammatory scores were assessed as continuous variables. The dependent variables were the dichotomous categories for the breast involution status. We defined having involuted breast; either complete lobular involution or predominant type 1/no type 3 lobules as our outcome of interest.

We hypothesized that higher tissue levels of pro-inflammatory markers would be associated with less involuted breasts (no or partial involution or predominant lobule types other than type 1 lobules), whereas, higher expression levels of anti-inflammatory markers would be associated with completely involuted breasts (higher prevalence of complete lobular involution and/or predominant type 1/no type 3 lobules). Univariate and multivariate generalized linear models were used to estimate the prevalence ratio and corresponding 95% CIs of our outcome of interest. *P*-value was based on score test for difference in prevalence.

We initially evaluated the potential confounding effects of age at mastectomy (years) as it was consistently associated with the ARLI across studies [10, 11, 20, 21]. The fully adjusted model included age at mastectomy (years), waist circumference (cm), as an index of adiposity, and menopausal status (pre- vs. postmenopausal) due to their association with the two measures of ARLI considered in our study. Inclusion of the body mass index (BMI) in the model rather than the waist circumference generated comparable results. All women were considered in the analyses and subsequent analyses stratified by menopausal status were performed. All statistical tests were two-sided and a *P* value less than 0.05 was considered significant. All statistical tests were carried out using the SAS software system 9.3 (SAS Institute Inc, Cary, NC).

## Results

Characteristics of the study population are listed in Table 1. The mean age at mastectomy was 52.6±7.8 years. Half of our study participants were postmenopausal (n = 82). On average, 57 lobules/woman (range, 6–389 lobules) were assessed while evaluating the predominant lobule type and 68 women (41.5%) were classified as having predominant type 1/no type 3 lobules. Among these, 49 women (72.1%) were parous. Of 119 parous women, 67 women (56.3%) had completely involuted breasts and 49 women (41.2%) had predominant type 1/no type 3 lobules. However, parity was not correlated to the ARLI in univariate analysis (r = 0.106, *P* = 0.177 and r = -0.009, *P* = 0.904 for the degree of lobular involution and the predominant lobule type, respectively). For BC risk factors, older age (r = 0.355, *P*<0.0001 and r = 0.458, *P*<0.0001 for the degree of lobular involution and the predominant lobule type, respectively) and higher waist circumference (r = 0.303, *P*<0.0001 and r = 0.241, *P*<0.002 for the degree of lobular involution and the predominant lobule type, respectively) were correlated with higher prevalence of complete lobular involution and predominant type1/no type 3 lobules in univariate analysis. Postmenopausal status was correlated with higher prevalence of complete lobular involution and predominant type1/no type 3 lobules in univariate analysis (r = 0.305, *P*<0.0001 and r = 0.421, *P*<0.0001 for the degree of lobular involution and the predominant lobule type, respectively). Postmenopausal women were more likely to have completely involuted breasts; 56 women (68.3%) had complete lobular involution and 51 women (62.2%) had predominant type 1/no type 3 lobules. More than half of women having complete involution (n = 57, 65.5%) and those having predominant type 1/no type 3 lobules (n = 44, 64.7%) had a waist circumference ≥86 cm, the median waist circumference for our study population. In addition, the two measures of the ARLI were moderately correlated as previously reported (r = 0.395, *P*<0.0001) [17]. There was no correlation between the use of oral contraception and the ARLI in age-adjusted analysis (r = 0.020 and *P* = 0.798 and r = 0.010, *P* = 0.895 for the degree of lobular involution and the predominant lobule type, respectively). Similarly, no correlation was observed between the use of hormone replacement therapy and the ARLI in age-adjusted analyses (r = 0.081 and *P* = 0.303 and r = -0.039, *P* = 0.625 for the degree of lobular involution and the predominant lobule type, respectively).

**Table 1:** Characteristics of the study population.

Characteristic	All women (n = 164)	Premenopausal (n = 82)	Postmenopausal (n = 82)
	mean ± SD	mean ± SD	mean ± SD
Age at mastectomy (years)	52.6 ± 7.8	46.8 ± 5.7	58.3 ± 4.9
Age at menarche (years)	12.6 ± 1.5	12.4 ± 1.3	12.8 ± 1.7
Age at first full-term pregnancy^a^ (years)	25.9 ± 4.1	26.2 ± 4.4	25.6 ± 3.8
Height (cm)	160.7 ± 6.3	161.6 ± 6.3	159.9 ± 6.2
Body mass index (kg/m^2^)	27.0 ± 5.7	26.4 ± 5.8	27.7 ± 5.5
Waist circumference (cm)	86.9 ± 12.7	83.8 ± 12.3	90.1 ± 12.4
Waist-to-hip ratio	0.81 ± 0.06	0.80 ± 0.06	0.83 ± 0.05
Number of live births^a^	2.1 ± 0.8	2.1 ± 0.8	2.0 ± 0.8
Alcohol consumption (drinks/week)	4.3 ± 4.6	4.6 ± 4.3	4.1± 4.9
	n (%)	n (%)	n (%)
Breastfeeding^a^	62 (37.8)	33 (40.2)	29 (35.4)
Oral contraceptive, ever used	157 (95.7)	79 (96.3)	78 (95.1)
Hormone replacement therapy, ever used	55 (33.5)	8 (9.8)	47 (57.3)
First-degree family history of breast cancer	34 (20.7)	13 (15.8)	21 (25.6)
Former or Current smoker	95 (57.9)	42 (51.2)	53 (64.6)
Educational level			
Elementary- secondary school completed	65 (39.6)	26 (31.7)	39 (47.6)
College completed	54 (32.9)	32 (39.0)	22 (26.8)
University completed	45 (27.4)	24 (29.3)	21 (25.6)
	n (%)	n (%)	n (%)
Two-category Quick score^b^, high level			
Pro-inflammatory markers, median			
IL-6, 2	95 (57.9)	56 (68.3)	39 (47.6)
TNF-α, 2	95 (57.9)	58 (70.7)	37 (45.1)
CRP, 2	85 (51.8)	57 (69.5)	28 (34.1)
COX-2 (epithelial expression), 6	103 (62.8)	61 (74.4)	42 (51.2)
COX-2 (stromal expression), 1	142 (86.6)	75 (91.5)	67 (81.7)
Leptin, 6	99 (60.4)	58 (70.7)	41 (50.0)
SAA1, 3	117 (71.3)	65 (79.3)	52 (63.4)
STAT3, 3	137 (83.5)	72 (87.8)	65 (79.3)
IL-8, 3	150 (91.5)	77 (93.9)	73 (89.0)
Anti-inflammatory markers, median			
TGF-β, 1	81 (49.4)	44 (53.7)	37 (45.1)
IL-10, 6	77 (46.9)	48 (58.5)	29 (35.4)
Lactoferrin, 3	104 (63.4)	60 (73.2)	44 (53.7)
Degree of lobular involution			
Complete involution	87 (53.0)	31 (37.8)	56 (68.3)
No or partial involution	77 (46.9)	51 (62.2)	26 (31.7)
Predominant lobule type			
Predominant type 1/no type 3 lobules	68 (41.5)	17 (20.7)	51 (62.2)
Other lobule types	96 (58.5)	65 (79.3)	31 (37.8)

Abbreviations: IL-6, interleukin 6; TNF-α, tumor necrosis factor-α; CRP, C-reactive protein; COX-2, cyclooxygenase 2; SAA1, serum amyloid A1; STAT3, signal transducer and activator of transcription 3; IL-8, interleukin 8; TGF-β, transforming growth factor-β; IL-10, interleukin 10.

^a^ Among parous women (n = 119, 59 and 60 for all, pre- and postmenopausal women, respectively).

^b^ Quick score was dichotomized into low and high expression using the median of each marker as cut-off.

As hypothesized, women with high levels of pro-inflammatory markers were less likely to have involuted breasts, either complete lobular involution or predominant type 1/no type 3 lobules compared to women with low tissue levels (Tables 2 and 3). Associations were significant for all pro-inflammatory markers and the prevalence of complete lobular involution, except for STAT3 in the age-adjusted models (*P* = 0.001, *P* = 0.0002, *P* = 0.004, *P* = 0.040, *P* = 0.037, *P*<0.0001, *P* = 0.001, *P* = 0.20 and *P* = 0.024 for IL-6, TNF-α, CRP, COX-2 (epithelial expression), COX-2 (stromal expression), leptin, SAA1, STAT3 and IL-8, respectively) (Table 2). Similar inverse associations were observed between the levels of COX-2 (epithelial expression), STAT3 or IL-8 with the prevalence of predominant type 1/no type 3 lobules in the age-adjusted models (*P* = 0.017, *P* = 0.080 and *P* = 0.052 respectively, Table 3). However, associations did not reach statistical significance for STAT3 and IL-8. Further adjustment did not substantially change any of the results.

**Table 2:** Association between the protein levels of inflammatory markers in normal breast tissue and the degree of lobular involution^a^.

Two-category quick score^b^	Degree of lobular involution								
	All women (n = 164)			Premenopausal women (n = 82)			Postmenopausal women (n = 82)		
Pro-inflammatory markers	N	Crude	Adjusted^c^	N	Crude	Adjusted^c^	N	Crude	Adjusted^c^
IL-6									
Low levels^d^	64	1.00	1.00	24	1.00	1.00	40	1.00	1.00
High levels	95	0.55 (0.41–0.75)	0.62 (0.47–0.84)	56	0.46 (0.27–0.80)	0.47 (0.27–0.81)	39	0.73 (0.53–1.00)	0.74 (0.54–1.01)
*P*^e^		<0.0001	0.001		0.012	0.014		0.047	0.047
*P*^e^^,^^f^			0.007			0.035			0.082
TNF-α									
Low levels^d^	65	1.00	1.00	22	1.00	1.00	43	1.00	1.00
High levels	95	0.50 (0.37–0.67)	0.57 (0.42–0.77)	58	0.35 (0.21–0.61)	0.36 (0.21–0.62)	37	0.74 (0.53–1.02)	0.75 (0.55–1.03)
*P*^e^		<0.0001	0.0002		0.001	0.001		0.060	0.069
*P*^e^^,^^f^			0.001			0.006			0.101
CRP									
Low levels^d^	76	1.00	1.00	23	1.00	1.00	53	1.00	1.00
High levels	85	0.52 (0.38–0.72)	0.63 (0.45–0.87)	57	0.33 (0.19–0.57)	0.33 (0.19–0.58)	28	0.92 (0.66–1.28)	0.96 (0.70–1.32)
*P*^e^		<0.0001	0.004		0.0004	0.0005		0.617	0.785
*P*^e^^,^^f^			0.007			0.001			0.757
COX-2 (epithelial expression)									
Low levels^d^	56	1.00	1.00	19	1.00	1.00	37	1.00	1.00
High levels	103	0.63 (0.47–0.84)	0.73 (0.55–0.98)	61	0.51 (0.30–0.88)	0.52 (0.30–0.90)	42	0.85 (0.62–1.15)	0.89 (0.66–1.20)
*P*^e^		0.002	0.040		0.038	0.044		0.293	0.441
*P*^e^^,^^f^			0.037			0.094			0.309
COX-2 (stromal expression)									
Low levels^d^	18	1.00	1.00	5	1.00	1.00	13	1.00	1.00
High levels	142	0.62 (0.46–0.84)	0.70 (0.53–0.93)	75	0.90 (0.30–2.74)	0.90 (0.28–2.86)	67	0.68 (0.53–0.87)	0.67 (0.53–0.85)
*P*^e^		0.017	0.037		0.860	0.866		0.011	0.008
*P*^e^^,^^f^			0.066			0.919			0.010
Leptin									
Low levels^d^	59	1.00	1.00	22	1.00	1.00	37	1.00	1.00
High levels	99	0.43 (0.32–0.58)	0.50 (0.36–0.68)	58	0.35 (0.21–0.61)	0.35 (0.20–0.61)	41	0.56 (0.40–0.79)	0.60 (0.43–0.84)
*P*^e^		<0.0001	<0.0001		0.001	0.001		0.0003	0.002
*P*^e^^,^^f^			<0.0001			0.001			0.001
SAA1									
Low levels^d^	38	1.00	1.00	15	1.00	1.00	23	1.00	1.00
High levels	117	0.55 (0.42–0.72)	0.62 (0.48–0.81)	65	0.44 (0.26–0.74)	0.44 (0.25–0.78)	52	0.70 (0.52–0.94)	0.70 (0.52–0.94)
*P*^e^		0.0002	0.001		0.016	0.016		0.025	0.023
*P*^e^^,^^f^			0.001			0.011			0.030
STAT3									
Low levels^d^	22	1.00	1.00	8	1.00	1.00	14	1.00	1.00
High levels	137	0.72 (0.51–1.00)	0.81 (0.60–1.09)	72	0.96 (0.37–2.48)	0.98 (0.38–2.53)	65	0.74 (0.55–0.98)	0.77 (0.59–1.00)
*P*^e^		0.090	0.200		0.939	0.097		0.063	0.076
*P*^e^^,^^f^			0.215			0.831			0.095
IL-8									
Low levels^d^	10	1.00	1.00	3	1.00	1.00	7	1.00	1.00
High levels	150	0.55 (0.42–0.71)	0.63 (0.47–0.84)	77	0.53 (0.22–1.24)	0.55 (0.22–1.32)	73	0.64 (0.54–0.76)	0.66 (0.52–0.84)
*P*^e^		0.010	0.024		0.336	0.359		0.008	0.022
*P*^e^^,^^f^			0.010			0.244			0.016
Anti-inflammatory markers									
TGF-β									
Low levels^d^	80	1.00	1.00	36	1.00	1.00	44	1.00	1.00
High levels	81	0.86 (0.64–1.15)	0.93 (0.70–1.23)	44	0.88 (0.49–1.57)	0.88 (0.49–1.57)	37	0.92 (0.68–1.25)	0.97 (0.72–1.30)
*P*^e^		0.303	0.614		0.658	0.669		0.593	0.831
*P*^e^^,^^f^			0.618			0.562			0.906
IL-10									
Low levels^d^	74	1.00	1.00	30	1.00	1.00	44	1.00	1.00
High levels	77	0.53 (0.38–0.75)	0.60 (0.43–0.84)	48	0.63 (0.35–1.12)	0.63 (0.35–1.13)	29	0.56 (0.37–0.87)	0.58 (0.38–0.87)
*P*^e^		0.0001	0.002		0.126	0.133		0.003	0.004
*P*^e^^,^^f^			0.002			0.116			0.004
Lactoferrin									
Low levels^d^	51	1.00	1.00	20	1.00	1.00	31	1.00	1.00
High levels	104	0.70 (0.52–0.95)	0.79 (0.60–1.06)	60	0.63 (0.36–1.13)	0.65 (0.36–1.19)	44	0.86 (0.62–1.20)	0.85 (0.62–1.17)
*P*^e^		0.030	0.130		0.161	0.198		0.384	0.320
*P*^e^^,^^f^			0.188			0.175			0.437

Abbreviations: IL-6, interleukin 6; TNF-α, tumor necrosis factor-α; CRP, C-reactive protein; COX-2, cyclooxygenase 2; SAA1, serum amyloid A1; STAT3, signal transducer and activator of transcription 3; IL-8, interleukin 8; TGF-β, transforming growth factor-β; IL-10, interleukin 10.

^a^ Degree of lobular involution was categorized as complete involution (≥ 75% TDLUs involuted) vs. no involution or partial involution (0–74% TDLUs involuted).

^b^ Quick score was dichotomized into low and high levels using the median of each marker as cut-off. Median of IL-6 = 2, TNF-α = 2, CRP = 2, COX-2 (epithelial expression) = 6, COX-2 (stromal expression) = 1, leptin = 6, SAA1 = 3, STAT3 = 3, IL-8 = 3, TGF-β = 1, IL-10 = 6, lactoferrin = 3.

^c^ Adjusted for age at breast surgery.

^d^ Referent category.

^e^ Score test of heterogeneity.

^f^ Adjusted for age at breast surgery and waist circumference. Analyses for all women were also adjusted for menopausal status.

**Table 3:** Association between the protein levels of inflammatory markers in normal breast tissue and the predominant lobule type^a^.

Two-category quick score^b^	Predominant lobule type								
	All women (n = 164)			Premenopausal women (n = 82)			Postmenopausal women (n = 82)		
Pro-inflammatory markers	N	Crude	Adjusted^c^	N	Crude	Adjusted^c^	N	Crude	Adjusted^c^
IL-6									
Low levels^d^	64	1.00	1.00	24	1.00	1.00	40	1.00	1.00
High levels	95	0.76 (0.52–1.11)	0.96 (0.68–1.34)	56	0.94 (0.37–2.42)	1.06 (0.43–2.60)	39	0.94 (0.66–1.35)	0.96 (0.68–1.35)
*P*^e^		0.166	0.793		0.904	0.897		0.748	0.809
*P*^e^^,^^f^			0.911			0.718			0.956
TNF-α									
Low levels^d^	65	1.00	1.00	22	1.00	1.00	43	1.00	1.00
High levels	95	0.62 (0.43–0.90)	0.82 (0.58–1.16)	58	0.38 (0.16–0.89)	0.43 (0.18–0.98)	37	1.03 (0.73–1.46)	1.05 (0.75–1.47)
*P*^e^		0.014	0.266		0.055	0.072		0.876	0.767
*P*^e^^,^^f^			0.467			0.118			0.630
CRP									
Low levels^d^	76	1.00	1.00	23	1.00	1.00	53	1.00	1.00
High levels	85	0.58 (0.40–0.85)	0.85 (0.59–1.23)	57	0.67 (0.28–1.64)	0.77 (0.32–1.86)	28	0.89 (0.61–1.30)	0.93 (0.64–1.35)
*P*^e^		0.005	0.380		0.417	0.577		0.541	0.700
*P*^e^^,^^f^			0.537			0.684			0.687
COX-2 (epithelial expression)									
Low levels^d^	56	1.00	1.00	19	1.00	1.00	37	1.00	1.00
High levels	103	0.50 (0.35–0.71)	0.65 (0.46–0.91)	61	0.24 (0.10–0.56)	0.26 (0.11–0.60)	42	0.85 (0.60–1.19)	0.89 (0.64–1.24)
*P*^e^		0.0003	0.017		0.009	0.009		0.339	0.492
*P*^e^^,^^f^			0.018			0.011			0.393
COX-2 (stromal expression)									
Low levels^d^	18	1.00	1.00	5	1.00	1.00	13	1.00	1.00
High levels	142	0.80 (0.49–1.33)	0.97 (0.63–1.48)	75	1.00 (0.16–6.11)	1.11 (0.20–6.27)	67	1.02 (0.64–1.63)	1.00 (0.66–1.53)
*P*^e^		0.436	0.882		1.000	0.899		0.938	0.984
*P*^e^^,^^f^			0.823			0.772			0.853
Leptin									
Low levels^d^	59	1.00	1.00	22	1.00	1.00	37	1.00	1.00
High levels	99	0.60 (0.41–0.86)	0.82 (0.58–1.16)	58	0.63 (0.26–1.53)	0.77 (0.32–1.84)	41	0.76 (0.54–1.09)	0.84 (0.59–1.18)
*P*^e^		0.008	0.274		0.354	0.574		0.130	0.304
*P*^e^^,^^f^			0.247			0.615			0.286
SAA1									
Low levels^d^	38	1.00	1.00	15	1.00	1.00	23	1.00	1.00
High levels	117	0.67 (0.45–0.98)	0.81 (0.57–1.17)	65	0.69 (0.26–1.85)	0.94 (0.37–2.37)	52	0.80 (0.56–1.15)	0.80 (0.56–1.15)
*P*^e^		0.063	0.281		0.512	0.898		0.250	0.250
*P*^e^^,^^f^			0.302			0.857			0.291
STAT3									
Low levels^d^	22	1.00	1.00	8	1.00	1.00	14	1.00	1.00
High levels	137	0.60 (0.41–0.87)	0.73 (0.53–1.00)	72	0.83 (0.23–3.00)	0.93 (0.29–2.99)	65	0.66 (0.49–0.90)	0.69 (0.52–0.93)
*P*^e^		0.033	0.080		0.798	0.910		0.025	0.033
*P*^e^^,^^f^			0.092			0.822			0.049
IL-8									
Low levels^d^	10	1.00	1.00	3	1.00	1.00	7	1.00	1.00
High levels	150	0.48 (0.33–0.70)	0.62 (0.42–0.91)	77	0.58 (0.11–3.08)	0.68 (0.16–2.98)	73	0.59 (0.49–0.71)	0.61 (0.47–0.80)
*P*^e^		0.022	0.052		0.629	0.683		0.007	0.017
*P*^e^^,^^f^			0.024			0.546			0.014
Anti-inflammatory markers									
TGF-β									
Low levels^d^	80	1.00	1.00	36	1.00	1.00	44	1.00	1.00
High levels	81	0.99 (0.68–1.43)	1.14 (0.83–1.58)	44	1.80 (0.69–4.71)	1.84 (0.72–4.68)	37	0.93 (0.66–1.32)	0.99 (0.71–1.37)
*P*^e^		0.948	0.417		0.207	0.180		0.701	0.943
*P*^e^^,^^f^			0.362			0.214			0.996
IL-10									
Low levels^d^	74	1.00	1.00	30	1.00	1.00	44	1.00	1.00
High levels	77	0.57 (0.38–0.87)	0.72 (0.50–1.06)	48	0.63 (0.24–1.60)	0.66 (0.25–1.71)	29	0.76 (0.51–1.14)	0.78 (0.54–1.13)
*P*^e^		0.007	0.087		0.348	0.403		0.164	0.180
*P*^e^^,^^f^			0.118			0.403			0.185
Lactoferrin									
Low levels^d^	51	1.00	1.00	20	1.00	1.00	31	1.00	1.00
High levels	104	0.68 (0.47–0.99)	0.82 (0.59–1.15)	60	1.00 (0.36–2.75)	1.34 (0.47–3.80)	44	0.77 (0.54–1.09)	0.85 (0.62–1.17)
*P*^e^		0.056	0.256		1.000	0.567		0.145	0.320
*P*^e^^,^^f^			0.347			0.569			0.122

Abbreviations: IL-6, interleukin 6; TNF-α, tumor necrosis factor-α; CRP, C-reactive protein; COX-2, cyclooxygenase 2; SAA1, serum amyloid A1; STAT3, signal transducer and activator of transcription 3; IL-8, interleukin 8; TGF-β, transforming growth factor-β; IL-10, interleukin 10.

^a^ Predominant lobule type was categorized as predominant type 1/no type 3 lobules vs. other lobule types.

^b^ Quick score was dichotomized into low and high levels using the median of each marker as cut-off. Median of IL-6 = 2, TNF-α = 2, CRP = 2, COX-2 (epithelial expression) = 6, COX-2 (stromal expression) = 1, leptin = 6, SAA1 = 3, STAT3 = 3, IL-8 = 3, TGF-β = 1, IL-10 = 6, lactoferrin = 3.

^c^ Adjusted for age at breast surgery.

^d^ Referent category.

^e^ Score test of heterogeneity.

^f^ Adjusted for age at breast surgery and waist circumference. Analyses for all women were also adjusted for menopausal status.

Similar associations were observed among pre- and postmenopausal women when assessed separately (Tables 2 and 3). Among premenopausal women, all pro-inflammatory markers were inversely associated with the prevalence of complete lobular involution in the age-adjusted models, except for COX-2 (stromal expression), STAT3 and IL-8. Likewise, among postmenopausal women, all pro-inflammatory markers were inversely associated with the prevalence of complete lobular involution in the age-adjusted models except for TNF-α, CRP, COX-2 (epithelial expression) and STAT3 (Table 2). Results remained significant in the fully adjusted models except for COX-2 (epithelial expression) among premenopausal women and for IL-6 among postmenopausal women. These inverse associations were less evident for the predominant lobule type in stratified analysis. Only COX-2 (epithelial expression) among premenopausal women and IL-8 among postmenopausal women were associated with higher prevalence of predominant type 1/no type 3 lobules in the age-adjusted model (Table 3). Results remained significant in the fully adjusted models.

When considering the pro-inflammatory score, we observed inverse associations with the prevalence of complete lobular involution among all women (*P*<0.0001), pre- (*P*<0.0001) and postmenopausal women (*P* = 0.008); and the prevalence of predominant type1/no type 3 lobules among all (*P* = 0.022) and premenopausal women (*P* = 0.019) in the age-adjusted models.

Contrary to our hypothesis, the levels of IL-10 was inversely associated with the prevalence of complete lobular involution among all and postmenopausal women in the age-adjusted models (*P* = 0.002 and *P* = 0.004, respectively) (Table 2). Further adjustment did not change the results. Similar inverse associations were observed for the anti-inflammatory score with the prevalence of complete lobular involution among all and premenopausal women in the age-adjusted models (*P* = 0.005 and *P* = 0.004, respectively). No significant associations were observed for the anti-inflammatory score with the prevalence of predominant type 1/no type 3 lobules (*P* = 0.078, *P* = 0.370 and *P* = 0.159 for all, pre- and postmenopausal women, respectively).

## Discussion

In this study, we report for the first time, significant inverse associations between the levels of inflammatory markers in breast tissue, mainly the pro-inflammatory ones, and the ARLI. In addition, the expression of all pro-inflammatory markers combined together was inversely associated with the prevalence of complete lobular involution and the predominant type 1/no type 3 lobules.

To our knowledge, we are the first to assess the association between the protein levels of inflammatory markers in normal breast tissue and the ARLI. Our findings are consistent with the previous observation that pro-inflammatory markers can promote sustained proliferation of mammary cells, DNA damage, angiogenesis, and induce an increase in local estrogen levels [5, 7, 22–24]. Conversely, anti-inflammatory markers are thought to exert an inhibitory activity on cellular proliferation [6, 25]. Moreover, it was previously shown that the anti-inflammatory marker TGF-β can induce the synthesis and deposition of extracellular matrix as well as the epithelial to mesenchymal transdifferentiation in mouse mammary gland [26, 27]. Inflammatory markers, most likely exert their effect on mammary cells by an autocrine or paracrine action, rather than endocrine effect [28, 29].

In previously published studies, the ARLI expressed either as the degree of lobular involution [10, 20]; the predominant lobule type [11]; the number of acini/lobule [12, 30]; the TDLU count/100 mm^2^ [12]; the lobule size [30]; or the median TDLU span [12] has been shown to be consistently inversely associated with BC risk. However, this inverse association may simply be explained by the regression of the epithelial structures susceptible to malignant transformation.

Despite the consistent association between the ARLI and reduced BC risk, the association between BC risk factors and the ARLI remains understudied. In our study, there was no correlation between parity and the two measures of the ARLI considered in our study. This is in line with previous studies reporting that neither the number of acini/lobule [31] or the predominant lobule type were related to parity [21]. In fact, hormonal changes during pregnancy stimulate the development of type 2 lobules into type 3 and 4 lobules, formed of milk secreting acini. Therefore, parous women are more likely to have type 3 lobules predominating in their breasts while breasts of nulliparous women consist predominantly of type 1 lobules with few type 2 and 3 lobules. Yet, it is generally assumed that upon completion of lobular involution both parous and nulliparous women have predominant type 1 lobule in their breasts. However, it was previously shown that type 3 lobules that had regressed back to type 1 lobules had decreased proliferative capacity with lower susceptibility for malignant transformation than morphologically indistinguishable type 1 lobules that never evolved into type 2 or 3 lobules [32, 33].

IL-10 is a pleiotropic immunosuppressive cytokine that can suppress the secretion of pro-inflammatory markers such as IL-6, TNF-α and IL-8. In our study, IL-10 was inversely associated with the prevalence of complete lobular involution particularly among postmenopausal women. The unexpected inverse association with the degree of lobular involution may be explained by its complex and controversial role in BC development. It is suggested that IL-10 can suppress the tumor development through the activation of natural killer, CD8^+^ and CD4^+^ T lymphocytes. On the other hand, IL-10 can also promote BC development by reducing the macrophage-antigen presenting capacity and inhibiting the production of many cytokines involved in the immunosurveillance process [34]. Not surprisingly, IL-10 was found to be highly produced by breast tumors compared to matched normal breast tissue [35].

Of interest, we have demonstrated associations between levels of inflammatory markers in normal breast tissue and the percent mammographic density (PMD), a known BC risk factor, in this population [18]. Women having high expression of pro-inflammatory markers IL-6, TNF-α, CRP or SAA1 had a higher PMD compared to those with low expression levels for the same markers. Conversely, women having higher expression of the anti-inflammatory marker, TGF-β, had less dense breasts compared to those having low TGF-β levels. Interestingly, the PMD was inversely associated with the ARLI [17]. More recently, the ARLI assessed as TDLU count/100 mm^2^ and median TDLU span was associated with PMD (*P* = 0.07 and *P* = 0.01, respectively) among 226 premenopausal women diagnosed with benign breast disease in multivariate analyses [36]. Of interest, the association between the circulating levels of IGF-I and TDLU counts observed among postmenopausal women were strongest among women with elevated mammographic density (*P*-interaction<0.0001) [7].

The strength of this study relies on the rich collection of epidemiological data available for study participants, the evaluation of the expression of multiple pro- and anti-inflammatory markers in normal breast tissue rather than their circulating levels, the application of TMA technique and the evaluation of all markers by one blinded reader. It was previously shown that circulating levels of inflammatory markers do not reflect their protein levels in breast tissue [37, 38]. Of note, we have previously demonstrated a fairly good concordance between the expression of inflammatory markers in TMA cores and corresponding measures in whole tissue sections (concordance = 81.5% (70–100%) and kappa = 0.62, 95% CI = 0.45–0.78) [18]. Moreover, the consideration of two measures of the ARLI, the degree of lobular involution and the predominant lobule type in the same study, providing both a qualitative and quantitative estimate, is also an advantage. Furthermore, the exhaustive evaluation of all lobules present on the selected slide contributed to obtaining a more accurate evaluation of the predominant lobule type than the more restrictive method of determining a fixed number of lobules per subject.

The main limitation of this study is the relatively small sample size that should be kept in mind when interpreting the results. Nevertheless, we were still able to detect statistically significant associations. Another limitation was the assessment of the ARLI in normal breast tissue obtained from BC patients, which may not be completely representative of normal breast tissue. However, there was no difference in types of lobules assessed in normal breast tissue located at >5.0 mm from the tumor and that obtained from women having benign breast diseases [39]. Moreover, there was no significant correlation between the two measures of ARLI considered in our study and the tumor grade. This suggests that the ARLI is independent of the adjacent pathology. In our study, we could not ascertain whether the detected levels of inflammatory markers in normal breast tissue influence BC risk or reflected the influence of the adjacent tumor because we used mastectomy specimens of women having BC. However, the recently reported absence of cancer field effects in normal breast tissue extracted from tumor-free mastectomy block may weaken this argument [40]. Equally important, the degree of lobular involution was found significantly lower among 1,115 women with benign breast disease of the multiple biopsy cohort compared to women enrolled in the Mayo benign breast disease cohort in age-adjusted analysis (*P*<0.001) [41]. In fact, all of our study participants have undergone a prior biopsy for diagnostic purposes. Although the biopsy performed prior to surgery might have caused an acute inflammatory reaction around the wound site, acute inflammation is short-lived and usually rapidly resolves by the elimination of the offending agent [42]. In our study, the time elapsed between the biopsy and the breast surgery (where study specimens were obtained) was ≥20 days for 98% of the participants. Therefore, any acute inflammation that could have evolved by the biopsy would have been resolved by the time of breast surgery and would not cause any confounding on the observed results. In addition, we could not include the menstrual phase at the time of mastectomy in any of the models due to lack of information. However, this should not have biased our results as the predominant lobule type was not associated with the menstrual phase in a previously published study [21]. In our study, there was no correlation between the time since menopause and the ARLI in age-adjusted analyses (r = 0.070, *P* = 0.377 and r = -0.110, *P* = 0.164 for the degree of lobular involution and the predominant lobule type, respectively).

## Conclusion

In summary, our findings suggest that the pro-inflammatory markers expressed by mammary cells may influence breast carcinogenesis by affecting the ARLI. Our findings have the potential to generate hypothesis about early events in BC development that provide the basis for future studies. Given the exploratory nature of this study, the limited sample size and the use of normal breast tissue obtained from women diagnosed with BC, larger studies are needed to elucidate these findings and to investigate whether the expression of inflammatory markers in breast tissue affects the age of onset and the rate of the ARLI.

## Abbreviations

ARLI: age-related lobular involution
BC: breast cancer
BMI: body mass index
COX-2: cyclooxygenase 2
CRP: C-reactive protein
DAB: diaminobenzidine
FFPE: formalin-fixed paraffin embedded
H&E: hematoxylin-eosin
HIER: Heat Induced Epitope antigen Retrieval
IL-10: interleukin 10
IL-6: interleukin 6
IL-8: interleukin 8
SAA1: serum amyloid A1
STAT3: signal transducer and activator of transcription 3
TDLU: terminal ductal lobular unit
TGF-β: transforming growth factor-β
TMA: tissue microarray
TNF-α: tumor necrosis factor-α
